# Supplementary material for: MetaRibo-Seq measures translation in microbiomes
Source: Nat Commun. 2020 Jun 29;11:3268. doi: 10.1038/s41467-020-17081-z (PMC7324362; doi:10.1038/s41467-020-17081-z)
Supplement: Supplementary file 10 — Supplementary Data 7 [file 41467_2020_17081_MOESM10_ESM.zip › File2/Confidence_High_Taxonomy/134517_out.krona.html]

Javascript must be enabled to view this page.

members
magnitude
magnitudeUnassigned
count
unassigned
taxon
rank

134517\_out

9

9
2
superkingdom

phylum
976
9

9
200643
class

9
171549
order

family
9
815

1
816
9
genus

SRS052576\_contig\_number\_contig-100\_11542.11543


SRS013965\_contig\_number\_contig-100\_1486.205305SRS143876\_contig\_number\_contig-100\_5297.5297
species
246787
2

1796613
1

SRS142599\_contig\_number\_2626
species


SRS019787\_contig\_number\_contig-100\_3930.3930
species
1
329854

species

SRS892882\_contig\_number\_contig-100\_17096.59334
457387
1

species

SRS971275\_contig\_number\_contig-100\_4918.236355
1
820

species

SRS1054691\_contig\_number\_contig-100\_19846.57879SRS143342\_contig\_number\_contig-100\_2002.118424
2
46506
